# The changes of endotracheal tube intracuff pressures after ear and head and neck surgery-related positions: a prospective observational study

**DOI:** 10.1016/j.bjorl.2020.05.005

**Published:** 2020-06-04

**Authors:** Hakan Kara, Dilek Hundur, Can Doruk, Dilan Buyuk, Gul Cansever, Emine Aysu Salviz, Emre Camci

**Affiliations:** a Istanbul University, Istanbul Faculty of Medicine, Department of Ear, Nose and Throat Surgery, Istanbul, Turkey; b Istanbul University, Istanbul Faculty of Medicine, Department of Anesthesiology and Reanimation, Istanbul, Turkey

**Keywords:** Endotracheal tube, Intracuff pressure, Ear surgery, Head and neck surgery, Surgery-related position

## Abstract

**Introduction:**

The cuff of an endotracheal tube seals the airway to facilitate positive-pressure ventilation and reduce subglottic secretion aspiration. However, an increase or decrease in endotracheal tube intracuff pressure can lead to many morbidities.

**Objective:**

The main purpose of this study is to investigate the effect of different head and neck positions on endotracheal tube intracuff pressure during ear and head and neck surgeries.

**Methods:**

A total of 90 patients undergoing elective right ear (Group 1: *n* = 30), left ear (Group 2: *n* = 30) or head and neck (Group 3: *n* = 30) surgery were involved in the study. A standardized general anesthetic was given and cuffed endotracheal tubes by the assistance of video laryngoscope were placed in all patients. The pilot balloon of each endotracheal tube was connected to the pressure transducer and standard invasive pressure monitoring was set to measure intracuff pressure values continuously. The first intracuff pressure value was adjusted to 18.4 mmHg (25 cm H_2_O) at supine and neutral neck position. The patients then were given appropriate head and neck positions before related-surgery started. These positions were left rotation, right rotation and extension by under-shoulder pillow with left/right rotation for Groups 1, 2 and 3, respectively. The intracuff pressures were measured and noted after each position, at 15th, 30th, 60th, 90th minutes and before the extubation. If intracuff pressure deviated from the targeted value of 20–30 cm H_2_O at anytime, it was set to 25 cm H_2_O again.

**Results:**

The intracuff pressure values were increased from 25 to 26.73 (25–28.61) cm H_2_O after left neck rotation (*p* = 0.009) and from 25 to 27.20 (25.52–28.67) cm H_2_O after right neck rotation (*p* = 0.012) in Groups 1 and 2, respectively. In Group 3, intracuff pressure values at the neutral position, after extension by under-shoulder pillow and left or right rotation were 25, 29.41 (27.02–36.94) and 34.55 (28.43–37.31) cm H_2_O, respectively. There were significant differences between the neutral position and extension by under-shoulder pillow (*p* < 0.001), and also between neutral position and rotation after extension (*p* < 0.001). However, there was no statistically significant increase of intracuff pressure between extension by under-shoulder pillow and neck rotation after extension positions (*p* = 0.033).

**Conclusion:**

Accessing the continuous intracuff pressure value measurements before and during ear and head and neck surgeries is beneficial to avoid possible adverse effects/complications of surgical position-related pressure changes.

## Introduction

Cuffed endotracheal intubation tubes (ETTs) are frequently and safely used for patients undergoing general anesthesia to secure the airway. The cuffs of the ETTs seal the extraluminal airway to facilitate positive-pressure ventilation and reduce subglottic secretion aspiration. As increase or decrease of ETT intracuff pressures (ICPs) can lead to many morbidities, therefore the volume of cuff must be set carefully. According to the guidelines, the target ICP should be between 20 and 30 cm H_2_O.^1^, ^2^ Overinflation of the ETT cuffs (high ICP) can cause laryngospasm, postoperative sore throat and stridor, laryngeal nerve damage, tracheal mucosal injury by compromising capillary perfusion, tracheal stenosis, tracheoesophageal fistula, tracheal bleeding and rupture.^3^, ^4^, ^5^, ^6^ On the other hand, underinflation (low ICP) can cause air leakage, aspiration and ventilation-associated pneumonia.^7^, ^8^

During the ear and head and neck surgery positions cuffed ETTs can displace and this can cause changes in ICP.^9^ With perioperative continuous monitorization, acute ICP rises and falls can be prevented and the abovementioned morbidities can be reduced.

There have been some reports that show the neck position change effects the ICP values from initial position to extension, flexion and lateral rotation (left/right).^9^, ^10^ These studies mainly investigated the ICP changes in patients who were provided healthcare in intensive care units. However; the effects of the routine combinations of neck positions on ICP during ear and head and neck surgeries have not been studied. This prospective observational study is designed to evaluate whether different head and neck positions in ear and head and neck surgeries would affect the ICPs at different positions and time points.

This study is reported according to the Strengthening the Reporting of Observational Studies in Epidemiology guidelines.^11^

## Methods

This prospective observational study was approved by our institution's (Istanbul University, Istanbul Faculty of Medicine) ethics committee (2019/156) and registered on ClinicalTrials.gov (NCT04037553). After written informed consent was obtained, 90 patients older than 18 years with American Society of Anesthesiologists (ASA) physical status I–III, who scheduled for elective ear or head and neck surgery under general anesthesia, were enrolled between February 2019 and May 2019, in the operating rooms of Department of Ear, Nose and Throat Surgery, Istanbul University, Istanbul Faculty of Medicine. Considering the study by Lizy et al.,^9^ a total of 30 patients per group was planned to be recruited for our study. Exclusion criteria were morbid obesity (Body Mass Index – BMI > 35), limited neck movements, previous history of radiotherapy or surgery to the head and neck area, nasotracheal intubation or peroperative tracheotomy requirements, respiratory tract infection and surgery planned for midline neck masses.

Standard anesthesia monitoring including the Electrocardiogram (ECG), pulse oximetry, noninvasive blood pressure and End-Tidal Carbon Dioxide (ETCO_2_) was applied to all patients. Midazolam (0.3 mg/kg), fentanil (1 mcg/kg), propofol (2–3 mg/kg) and rocuronium (0.6 mg/kg) were administered to all patients for standard general anesthesia induction. Video laryngoscope (C-MAC®, KARL STORZ, Tuttlingen, Germany) – assisted endotracheal intubation of all female and male patients was performed using a cuffed ETT (Bıçakçılar AŞ, Istanbul, Turkey) that has an inner diameter of 7.5 or 8 mm according to the patient's gender. Correct cuff position was confirmed by visualization of ETCO_2_ and auscultation of bilateral breath sounds. Each patient's ETT cuff was inflated with a 2 mL syringe until no air leak was heard. The endotracheal intubation tube of every patient was taped to the contralateral side of his/her mouth depending on the site of the surgery. Patients were ventilated by using a tidal volume of 6–8 mL/kg with the respiratory rate of 12–14 cycle/min. General anesthesia was maintained with sevoflurane (1%–3%) in a mixture of oxygen (40%) and air (60%) without the administration of Nitrous Oxide (N_2_O). End-tidal CO_2_ levels were kept between 30 and 35 mmHg. Then, the pilot balloon of each patient's cuffed ETT was connected to the disposable pressure transducer set, and standard Invasive Pressure Monitoring Setup (IPMS) was prepared and calibrated for all patients to measure and monitorize ICP as Gopalakrishnan et al.^12^ reported as a validated method.

The patients were allocated to 3 groups according to their surgery types. Groups 1 and 2 included the patients who underwent right and left ear surgeries, respectively. Group 3 included all patients undergoing head and neck surgery without considering the surgical site.

**Figure 1:**
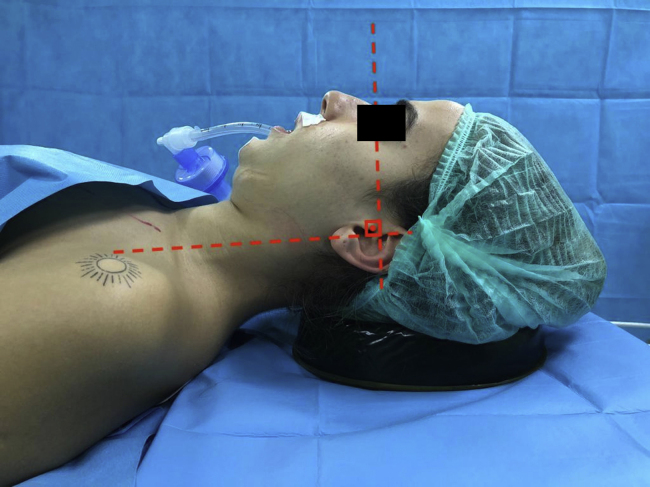
The initial head and neck (neutral) positions of all patients while they were in supine position. Gel pillow (height: 3 cm) was placed under patients’ heads and the two imaginary lines (1) between external ear canal to the top of the shoulder and (2) the external ear canal to the superior orbital margin were adjusted to be vertical.

All surgeries were performed while the patients were in supine position. After the general anesthesia induction, the patients were given a neutral position defined by Komasawa et al.^13^ in order to standardize their initial head and neck positions. Gel pillow (height: 3 cm) was placed under patients’ heads and the two imaginary lines (1) between external ear canal to the top of the shoulder and (2) the external ear canal to the superior orbital margin were adjusted to be vertical (Fig. 1).

The first ICP was measured at a neutral position just after taping the tube and monitoring the 3 consecutive respiration cycles. If high, it was lowered to 18.4 mmHg which is the mean value of suggested pressures (ideal ICP of 25 cm H_2_O was aimed to be obtained by multiplying 18.4 mmHg with 1.36).^1^, ^2^

After neutral measurements, appropriate head and neck positions were applied to the patients before surgery started. Left or right neck rotation (approximately 60–70 degree to the opposite side) was applied to Group 1 or 2 in conformity with the ear operation site and ICP value was documented following 3 respiration cycles. Besides the underhead gel pillow, another one with the height of 4.5 cm was placed under the shoulders of Group 3 patients to extend the neck. After waiting for 3 respiration cycles, the ICP was noted. Then, right or left neck rotation was applied depending on the operation site (approximately 60–70 degree to the opposite side). Following 3 respiration cycles, the ICP was documented again. Additionally, ICP values were monitored continuously in all patients during the surgeries, and documented at 15th, 30th, 60th and 90th minutes at related positions and just before extubation at neutral position. At anytime, if ICP value fell below 14.7 mmHg (20 cm H_2_O) or rose above 22 mmHg (30 cm H_2_O), ICP was adjusted to 18.4 mmHg (25 cm H_2_O) again.

### Statistics

Power analysis was computed by using G*Power.^14^ The sample size was calculated separately for different neck positions based on the results of a previous study.^9^ The differences in mean ICP values between neutral position and left rotation, right rotation and extension were 4.5, 6.5 and 9.1 cm H_2_O, while the differences in standard deviation between positions were 4.8, 9 and 9.5, respectively. To achieve a power of 0.95 and an *α* of 0.05 with two-tailed test, required patient numbers were 17, 27 and 17 for left and right rotations and extension, respectively. We decided to recruit 30 patients for each group to compensate the possible dropouts.

Statistical Package for the Social Sciences (SPSS) version 22 (IBM Corp., Armonk, NY, USA) was used to perform further statistical analysis. A value of *p* < 0.05 was considered as statistically significant. Descriptive analysis was performed to report demographic data. The Shapiro–Wilk test was used to evaluate the normality of the data. The *p*-value of average ICPs at neutral position were <0.001 for all three groups, which displayed anormal distribution of the data. Since, we have set all cuff pressure to 25 cm H_2_O on purpose, it was an expected result. Therefore, we chose non-parametric tests for further analysis. Non-parametric related-samples Wilcoxon signed-rank test was used to compare the average ICP at neutral position with defined position in patients who underwent ear surgery. Non-parametric related-samples Friedman's two-way analysis of variance was tested to show differences of the mean value of ICP between neutral and defined positions in patients who underwent head and neck surgery. Then the post hoc analysis was performed with Wilcoxon signed-rank test to examine where the difference actually occurred. Post hoc analysis with Wilcoxon signed-rank tests was conducted with a Bonferroni correction applied, resulting in a significance level set at *p* < 0.017.

## Results

**Figure 2:**
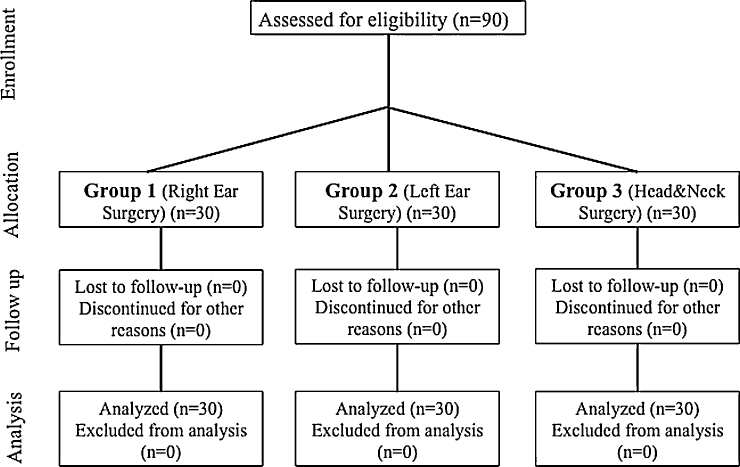
Strengthening the Reporting of Observational Studies in Epidemiology Diagram of Groups 1, 2 and 3. Group 1: patients underwent right ear surgery, Group 2: patients underwent left ear surgery, and Group 3: patients underwent head and neck surgery.

A total of 90 patients who were scheduled for elective ear or head and neck surgery were eligible and allocated to 1 of the 3 groups according to their surgery types. None of the patients were excluded from the study. Thirty patients in each group completed the study (Fig. 2).

The demographic data, anesthesia and surgery properties of all patients, ETT ICP measurements and their position-related changes are shown in Table 1.

**Table 1:** Patient demographics, surgery types, endotracheal tube (ETT) intracuff pressure (ICP) measurements, surgery and anesthesia durations.

	Group 1	Group 2	Group 3
*Age (yr)*^a^	42.80 ± 12.92	36.43 ± 12.25	46.97 ± 16
*BMI (kg/m*^*2*^*)*^a^	25.70 ± 3.71	25.94 ± 4.40	26.89 ± 4.25
*Gender (F/M)*	18/12	18/12	16/14

*Surgery types*			
Tympanoplasty			
Mastoidectomy	13	17	
Stapes surgery	8	4	
Atticotomy	4	4	
Infratemporal fossa approach	4	2	
Ventilation tube insertion	1		
Cochlear implantation		2	
Cervical lympadenopatyh biopsy		1	11
Parotidectomy			10
Glomus caroticum excision			3
Branchial cyst excision			3
Neck dissection			1
Submandibular gland excision			1
Lipoma excision			1

*ETT ICPs (cm H*_*2*_*O)*^b^			
Neutral position	25.00	25.00	25.00
Left rotation	26.73 (25–28.61)		
Right rotation		27.20 (25.52–28.67)	
Neck extension			29.41 (27.02–36.94)
Lateral rotation after extension			34.55 (28.43–37.31)

*Surgery duration (min)*^b^	100 (70–141.25)	97.5 (73.75–130)	55 (45–115)
*Anesthesia duration (min)*^b^	127.5 (90–165)	120 (88.75–150)	75 (60–137.5)

Group 1: patients underwent right ear surgery; Group 2: patients underwent left ear surgery; Group 3: patients underwent head and neck surgery.

BMI, Body Mass Index; F, female; M, male.

a Data are presented as mean ± SD.

b Data are presented as median (first through third quartile).

**Figure 3:**
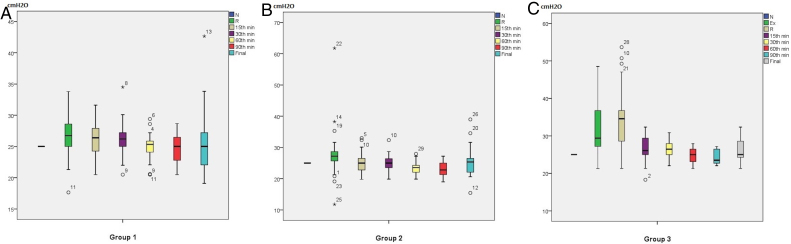
Endotracheal tube (ETT) intracuff pressure (ICP) measurements (cm H_2_O): A, Group 1: at Neutral (N), left neck Rotation (R) after 3 respiration cycles, at 15th, 30th, 60th, 90th minutes and just before extubation (Final) at neutral position. B, Group 2: at Neutral (N), right neck Rotation (R) after 3 respiration cycles, at 15th, 30th, 60th, 90th minutes and just before extubation (Final) at neutral position. C, Group 3: at Neutral (N), Extension (Ex), left or right neck Rotation (R) depending on the operation site after 3 respiration cycles, at 15th, 30th, 60th, 90 ^th^ minutes and just before extubation (Final) at neutral position.

Endotracheal tube ICPs of Groups 1 and 2 at Neutral (N), left (Group 1) or right (Group 2) neck Rotations (R) after 3 respiration cycles, at 15th, 30th, 60th, 90th minutes and just before extubation (Final) at neutral position were shown in Fig. 3A and B.

The median ICP value was increased from 25 to 26.73 (25–28.61) cm H_2_O when left rotation neck position was applied to Group 1 patients (*p* = 0.009) (Table 1, Fig. 3A). Among the 30 patients, 22 patients’ ICPs were increased. The number of patients whose ICP values decreased was 6. Two patients’ ICP values remained same. In six patients, the ICP values got outside the targeted-interval. Five of them were above 30 cm H_2_O and one was below 20 cm H_2_O (17.65 cm H_2_O). The maximum ICP value measured in this group was 33.80 cm H_2_O.

The median ICP value was increased from 25 to 27.20 (25.52–28.67) cm H_2_O when right rotation neck position was applied to Group 2 patients (*p* = 0.012) (Table 1, Fig. 3B). Twenty-three patients’ ICP values were increased, while decreased ICP values were obtained from 6 patients. The ICP remained same in only one patient. After the right neck rotations, the ICP values were above 30 cm H_2_O in 6 patients and below 20 cm H_2_O in 2 patients. The maximum ICP value reached 61.76 cm H_2_O, while the minimum ICP was obtained as 11.76 cm H_2_O in this group.

Totally, ICP increased in 75% (45/60) of the ear surgery patients and exceeded the maximum pressure level (30 cm H_2_O) in 18.3% (11/60) of patients after the left/right rotation.

Endotracheal tube ICPs of Group 3 at Neutral (N), Extension (Ex), left or right neck Rotation (R) depending on the operation site after 3 respiration cycles, at 15th, 30th, 60th, 90th minutes and just before extubation (Final) at neutral position were shown in Fig. 3C.

In Group 3, there was a difference in ICP values depending on head and neck position while preparing to surgery (*p* < 0.001). Median ICP values for the neutral position, extension by under-shoulder pillow and rotation after extension were 25, 29.41 (27.02–36.94) and 34.55 (28.43–37.31) cm H_2_O, respectively (Table 1, Fig. 3C). There were significant differences between the neutral position and extension by under-shoulder pillow (*p* < 0.001), and also between neutral position and rotation after extension (*p* < 0.001). However; there was no statistically significant increase of ICP between extension by under-shoulder pillow and rotation after extension positions (*p* = 0.033).

Among Group 3 patients, ICP was increased in 26 patients (86.7%) and decreased in 4 after extension position. In addition; after the combination of neck extension and rotation, ICP measurement increase and decrease were seen in 17 (56.7%) and 9 (30%) patients, respectively. Four patients’ ICP values maintained the same level after additional neck rotation. Combined position ICPs were increased in 26 patients compared to neutral position ICPs. However, it decreased in 3 patients and remained the same in 1 patient. In 17 of 26 patients, ICP values rose above 30 cm H_2_0. The maximum measured value was 53.68 cm H_2_O.

During the whole general anesthesia period (including surgery-related position, surgery duration and extubation), the ICP values were corrected 0.6, 1.3 and 1.5 times in average using the pilot balloons of cuffed ETTs in Groups 1, 2 and 3, respectively as it exceeds the targeted-interval level of 20–30 cm H_2_O.

## Discussion

Our results showed that there have been significant differences in ICP values between neutral and each surgery-related position. Although incremental changes were observed in 71/90 (79%) patients and ICP values exceeded the maximum ICP level of 30 cm H_2_O in 28/90 (31.1%) patients after final positions, there have also been decrements.

In daily clinical otolaryngology practice, patients’ head and neck positions such as rotations and extensions are in use both to observe the surgical field better and to avoid vital anatomical structures that might be the reason of perioperative surgical adverse events and complications. During these positioning periods, keeping the ETT ICP values within the optimum interval is also vital to prevent possible airway-related events.^3^, ^4^, ^5^, ^6^, ^7^, ^8^

Variations in ICP due to environmental factors, interventions and patient-related factors have already been shown in the literature. Environmental factors such as high altitude^15^ (transport by helicopter), interventions such as positive-pressure ventilation^16^ or ventilation with nitrous oxide^17^ and any anatomical variations^18^, ^19^ or pathological processes including constriction of the airway cause an increase in ICP. On the other hand, use of muscle relaxing agents might cause a decrease in ICP.^20^ Furthermore, it has been showed in other studies that several changes in head and neck positions can significantly affect the ICP and may sometimes lead to tube displacement.^4^, ^9^, ^10^, ^21^

Brimacombe et al.^21^ determined increased ICP values after rotated, extended and flexed positions of the head in their 10 anesthetized patients, although their starting pressure was higher than the recommended target pressure (40.8 cm H_2_O). They emphasized that the rotation caused the greatest increase in tracheal mucosal pressure than other two positions. De Godoy et al.^22^ showed cuff pressure rise in 50.7% of their measurements in mechanically ventilated patients after 35° semirecumbent to lateral positioning and back, while using target ICP interval of 24.5–29.9 cm H_2_O. The rise in pressures obtained after changing the position from supine to prone and also occurred by head flexion and extension in supine and prone positions were demonstrated in study performed by Kim et al.^4^ Another study comparing the ICPs of cuffed ETTs with different properties and shapes after various positions showed significant ICP changes after flexion with both cuff types and significant ICP changes after extension with only High Volume Low Pressure (HVLP) cuff type.^13^ In addition to these increases; Lizy et al.^9^ reported that 40.6% of their 192 measurements (after 16 position changes in their 12 patients) were greater than the upper target limit of 30 cm H_2_O. With respect to all these clinical investigations, despite ICP changes in different positions were examined, the overall data mainly demonstrate the significant rise in ICP values after positioning. In our study, compared to the neutral position we similarly found the ICP rise after each change of a patient's neck rotation and extension position. Moreover, our results showed ICPs higher than 30 cm H_2_O in totally 11 patients after left and right rotations, and 17 patients after the combination of neck extension and rotation positions.

Increases in ICPs and especially values higher than 30 cm H_2_O are common and clinically important as they may compromise the mucosal capillary perfusion and lead to tracheal injury, especially in long lasting cases and in Intensive Care Units (ICUs).^4^, ^9^, ^13^, ^21^, ^22^, ^23^, ^24^, ^25^, ^26^ These results including tracheal stenosis (estimated incidence of 4%–12% within 24 days of intubation) or fistula formation may occur in chronic situations; however it is important to be aware of detrimental effects and prevent acute injuries.^27^ These risks are clearly associated with high ICPs on the tracheal wall; however as anesthetists, ICU doctors and otolaryngologists, we do not really follow patients’ cuff pressures continuously with reliable techniques.

In contrast to abovementioned increased cuff pressure findings, De Godoy et al.^22^ showed ICP decreases only in 5% of the patients and Lizy et al.^9^ did not even report any measurements less than the lower target limit of 20 cm H_2_O. Our data indicate that ICPs decreased in totally 23.3% of patients and only 3 of them were below 20 cm H_2_O after only left or right neck rotations. Even though increase in ICP is a more expected consequence of head and neck positions, clinicians should also be aware that ICP decrease resulting in air leakage, aspiration and ventilation-associated pneumonia is also possible.^7^, ^8^

In our study, although patients with morbid obesity, limited neck movements, previous history of radiotherapy or surgery to the head and neck area, respiratory tract infection and long lasting surgery planned for midline neck masses with/without neck dissection to avoid the external pressure on the trachea were all excluded, ICP measurements exceed the targeted-interval level of 20–30 cm H_2_O ICP values a couple of times in each group and were corrected. These results indicate the importance of continuous ICP measurements once more and emphasize the requirements of corrections.

Automatic devices that monitorize and adjust the exceeded ICPs have also been developed. Those devices maintain the pressure within the targeted interval level of 20–30 cm H_2_O and are especially helpful for surgeries with different head and neck positions, surgeries themselves on the neck, long- lasting cases and long- term intubated ICU patients.^28^, ^29^

Our study has limitations. Firstly, automatic devices could have been used for the ICP monitoring. However, as the device we have automatically makes adjustments in ICP if it is not within the targeted interval, we did not prefer to use such a device for recording the ICP values accordingly. Secondly, the displacement of the ETTs was not assessed by fiberoptic bronchoscopy after position changing. Thirdly, this present study has been performed in patients undergoing elective surgeries that last 30 min to 3 h in contrast to the long-term hospitalized ICU patients. Although we are aware of the intraoperative rises in ICP values, we have not followed the patients postoperatively and did not assess the clinical consequences in this study.

## Conclusion

Our results showed significant increases in ICP levels due to rotation and extension positions that are commonly-used maneuvers by otolaryngologists. The continuous ICP value measurements before and during ear and head and neck surgeries are beneficial to avoid possible adverse effects/complications of long lasting surgical position-related pressure changes.

## Ethics committee approval

Istanbul University, Istanbul Faculty of Medicine – no. 2019/156.

ClinicalTrials.gov identifier: NCT04037553.

## Conflicts of interest

The authors declare no conflicts of interest.

## References

[1] Lorente L., Blot S., Rello J. (2007). Evidence on measures for the prevention of ventilator-associated pneumonia. Eur Respir J.

[2] (2005). Guidelines for the management of adults with hospital-acquired, ventilator-associated, and healthcare-associated pneumonia. Am J Respir Crit Care Med.

[3] Dobrin P., Canfield T. (1977). Cuffed endotracheal tubes: mucosal pressures and tracheal wall blood flow. Am J Surg.

[4] Kim D., Jeon B., Son J.S., Lee J.R., Ko S., Lim H. (2015). The changes of endotracheal tube cuff pressure by the position changes from supine to prone and the flexion and extension of head. Korean J Anesthesiol.

[5] Davies J.D., May R.A., Bortner P.L., Hess D.R., MacIntyre N.R., Mishoe S.C., Galwin W.F., Adams A.B. (2012).

[6] El-Boghdadly K., Bailey C.R., Wiles M.D. (2016). Postoperative sore throat: a systematic review. Anaesthesia.

[7] Safdar N., Crnich C.J., Maki D.G. (2005). The pathogenesis of ventilator-associated pneumonia: its relevance to developing effective strategies for prevention. Respir Care.

[8] Rello J., Soñora R., Jubert P., Artigas A., Rué M., Vallés J. (1996). Pneumonia in intubated patients: role of respiratory airway care. Am J Respir Crit Care Med.

[9] Lizy C., Swinnen W., Labeau S., Poelaert J., Vogelaers D., Vandewoude K. (2014). Cuff pressure of endotracheal tubes after changes in body position in critically ill patients treated with mechanical ventilation. Am J Crit Care.

[10] Kim J.T., Kim H.J., Ahn W., Kim H.S., Bahk J.H., Lee S.C. (2009). Head rotation, flexion, and extension alter endotracheal tube position in adults and children. Can J Anesth.

[11] Von Elm E., Altman D.G., Egger M., Pocock S.J., Gøtzsche C., Vandenbroucke J.P. (2007). Policy and practice the Strengthening the Reporting of Observational Studies in Epidemiology (STROBE) statement: guidelines for reporting observational studies. Bull World Health Organ.

[12] Gopalakrishnan S., Barry N., Rice J., Tobias J.D. (2013). Cuffed endotracheal tubes in infants and children: a technique to continuously measure the intracuff pressure. Int J Pediatr Otorhinolaryngol.

[13] Komasawa N., Mihara R., Imagawa K., Hattori K., Minami T. (2015). Comparison of pressure changes by head and neck position between high-volume low-pressure and taper-shaped cuffs: a randomized controlled trial. Biomed Res Int.

[14] Faul F., Erdfelder E., Lang A.-G., Buchner A. (2007). G*Power 3: a flexible statistical power analysis program for the social, behavioral, and biomedical sciences. Behav Res Methods.

[15] Bassi M., Zuercher M., Erne J.J., Ummenhofer W. (2010). Endotracheal tube intracuff pressure during helicopter transport. Ann Emerg Med.

[16] Bernhard W.N., Yost L., Joynes D., Cothalis S., Turndorf H. (1985). Intracuff pressures in endotracheal and tracheostomy tubes: related cuff physical characteristics. Chest.

[17] Nguyen Tu H., Saidi N., Lieutaud T., Bensaid S., Menival V., Duvaldestin P. (1999). Nitrous oxide increases endotracheal cuff pressure and the incidence of tracheal lesions in anesthetized patients. Anesth Analg.

[18] Mehta S., Myat H.M. (1984). The cross-sectional shape and circumference of the human trachea. Ann R Coll Surg Engl.

[19] Mackenzie C.F., McAslan T.C., Shin B., Schellinger D., Helrich M. (1978). The shape of the human adult trachea. Anesthesiology.

[20] Girling K.J., Bedforth N.M., Spendlove J.L., Mahajan R.P. (1999). Assessing neuromuscular block at the larynx: the effect of change in resting cuff pressure and a comparison with video imaging in anesthetized humans. Anesth Analg.

[21] Brimacombe J., Keller C., Giampalmo M., Sparr H.J., Berry A. (1999). Direct measurement of mucosal pressures exerted by cuff and non-cuff portions of tracheal tubes with different cuff volumes and head and neck positions. Br J Anaesth.

[22] De Godoy A.C.F., Vieira R.J., De Capitani E.M. (2008). Endotracheal tube cuff pressure alteration after changes in position in patients under mechanical ventilation. J Bras Pneumol.

[23] Galinski M., Tréoux V., Garrigue B., Lapostolle F., Borron S.W., Adnet F. (2006). Intracuff pressures of endotracheal tubes in the management of airway emergencies: the need for pressure monitoring. Ann Emerg Med.

[24] Chopra M., Jones L., Boulanger C., Benger J., Higginson I., Williamson D. (2010). Prospective observational measurement of tracheal tube cuff pressures in the emergency department. Emerg Med J.

[25] Braz J.R., Navarro L.H., Takata I.H., Nascimento Júnior P. (1999). Endotracheal tube cuff pressure: need for precise measurement. Sao Paulo Med J.

[26] Rosero E.B., Ozayar E., Eslava-Schmalbach J., Minhajuddin A., Joshi G.P. (2018). Effects of increasing airway pressures on the pressure of the endotracheal tube cuff during pelvic laparoscopic surgery. Anesth Analg.

[27] Whited Re. (1984). A prospective study of laryngotracheal sequelae in long-term intubation. Laryngoscope.

[28] Nseir S., Zerimech F., Fournier C., Lubret R., Ramon P., Durocher A. (2011). Continuous control of tracheal cuff pressure and microaspiration of gastric contents in critically ill patients. Am J Respir Crit Care Med.

[29] Valencia M., Ferrer M., Farre R., Navajas D., Badia J.R., Nicolas J.M. (2007). Automatic control of tracheal tube cuff pressure in ventilated patients in semirecumbent position: a randomized trial. Crit Care Med.

